# MatGel: A MATLAB program for quantitative analysis of 2D polyacrylamide electrophoresis (2D-PAGE) protein gel images

**DOI:** 10.1016/j.mex.2022.101930

**Published:** 2022-11-21

**Authors:** Alka Tiwari, W. Paul Williams, Xueyan Shan

**Affiliations:** a Department of Biochemistry, Molecular Biology, Entomology and Plant Pathology, Mississippi State University, MS 39762, USA; b USDA-ARS, Corn Host Plant Resistance Research Unit, MS 39762, USA

**Keywords:** Bioinformatics, Proteomics, TopHat filter, Watershed segmentation

## Abstract

Two-dimensional polyacrylamide gel electrophoresis (2D-PAGE) is widely used in proteomics studies. Hundreds of proteins extracted from a biological sample can be separated and visualized on a 2D-PAGE gel. The interpretation of protein expression levels relies on the comparison of areas and intensities of the corresponding protein spots in 2D-PAGE gel images. However, determination of protein spot areas by the manual selection method is time-consuming and error-prone. The purpose of this research is to develop a highly automated program for the simultaneous detection and quantification of protein spots across a large number of 2D-PAGE protein gel images by using MATLAB image processing toolbox. This program will enhance the potential of using 2D-PAGE technique as a high throughput quantitative protein expression study tool. We developed MatGel, a simple and efficient program for protein spot area detection and intensity quantification from 2D-PAGE protein gel images. MatGel can detect and output the areas and mean intensities of corresponding protein spots across a large number of 2D-PAGE gel images simultaneously. Users also have options to adjust preferences at each step of image analysis. Basic knowledge with MATLAB programming language is required to run the program.

• We developed MATLAB program MatGel to automate the determination of protein spots on 2D-PAGE protein gel images.
• MatGel can analyze a large number of 2D-PAGE gel images simultaneously to minimize human errors.
• MatGel is flexible and easy to use.

## Graphical abstract

**Figure:**
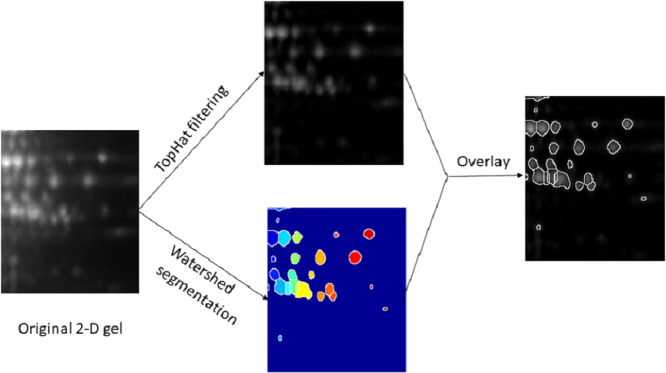

Specifications table

**Table:** 

Subject area:	Bioinformatics
More specific subject area:	Proteomics
Name of your method:	MatGel
Name and reference of original method:	Not applicable
Resource availability:	Two Matlab program code files MatGel_align_crop.m and MatGel_segmentation.m and instructions are provided in the supplementary material.

## Method details

### Background information

The two-dimensional polyacrylamide gel electrophoresis (2D-PAGE) protein gel technique is a powerful tool that allows the separation and visualization of hundreds of proteins on a polyacrylamide gel [1]. 2D-PAGE method generates a wealth of image data that must be analyzed by computational methods. Several software packages are commercially available for protein spot detection and analysis [2]. However, to use these software packages users need to evaluate the accessibility and expense of using their associated instrumentation and the considerable effort that is required for manual operations in the image analysis process [3]. Users must perform substantial manipulation at many stages including the manual selection of protein spots, manual adjustment of threshold values on individual gels, and manual output of results with the lack of statistical tools suitable for their needs [4]. These intensive and time-consuming user interventions can potentially enlarge the sample variations in the experimental procedure of 2D-PAGE [5]. Studies have showed that software induced variance can be much higher than the inherit variance present in the experimental data [6].

MatGel is a program developed in MATLAB programming language. It requires the additional MATLAB Digital Image Processing Toolbox© (MathWorks, Natick, MA, USA). The workflow of image analysis contains six main steps: (1) preparation of protein gel images, (2) alignment of gel images to a standard gel image using appropriate image registration methods, (3) mean gel image construction, (4) morphological operations for edge detection and segmentation to identify the protein spot areas, (5) the output of areas and mean intensities of detected protein spots, and (6) statistical analysis of the data. The construction of a mean gel image from all aligned and cropped gel images is an essential step for the identification of equivalent areas of corresponding protein spots and the minimization of errors in extracting quantitative data (pixel intensities) across multiple gel images. The Matlab programs of MatGel is provided in the supplemental material. Freely available MatGel will become a useful research tool of biochemists, allowing for the code modification for customized analysis to meet user's specific needs.

### Preparation of protein gel images

2D-PAGE protein gel images can be taken by using any digital gel imaging systems that match with the protein gel staining dyes. Images can be saved in an image file format such as tiff, jpeg, bmp, or jpg.

### Alignment and cropping of images

MatGel comprises two script files written in MATLAB programming language (MatGel_align_crop.m and MatGel_segmentation.m, Supplementary Material). To run these MatGel script files, a copy of MATLAB (MathWorks, Natick, MA, USA) and the MATLAB Image Processing Toolbox are required.

The first step is to create a new directory and name it as MatGel. Put all the 2D-PAGE gel images and the two MatGel script files in the MatGel directory. Launch MATLAB and make sure the MatGel directory is in the path of MATLAB.

Load and run MatGel_align_crop.m file. It brings up a message box ‘please select the standard gel image’. Clicking on ‘ok’ will cause the MatGel directory window to show up. Choose one 2D-PAGE gel image as the standard image. The same standard image will be used for the whole experiment. Follow the instruction from the next message box and select a gel image to be aligned with the standard gel image. The MATLAB GUI window for the control point selection will be launched at this point. Select two common protein spots present in all the 2D-PAGE images and use them as the control points to align a gel image to the standard gel image. After the image alignment, select a suitable size for image cropping. The selection of size for image cropping is only requested one time. All images afterwards will be automatically cropped to the same size. Repeat the image alignment process until all the 2D-PAGE gel images in the experiment are aligned and cropped one by one along with the standard gel image. The alignment step is the only step that involves user's intervention regarding the quality of data. It is important to assure the accuracy in the alignment of the control point protein spots between gel images. After all the alignments are done, two new folders will be created in the MatGel directory: one is for the storage of all aligned gel images and the other is for all the aligned and cropped gel images.

### Mean gel image construction, edge detection and segmentation, and output of the areas and mean intensities

This whole procedure is highly automated. It only takes a few seconds to get an output Excel file containing all the quantitative data, despite the number of 2D-PAGE gel images included in the experiment. The MatGel segmentation.m file is used for this with little user intervention required. At the start of the program, the user is asked to select the folder containing all the aligned and cropped gel images. The program then automatically reads all images from the folder and generates a mean gel image. The user can then define the region of interest (ROI) from the mean gel image to be used for edge detection and segmentation. The program detects edges for protein spots from the mean gel image and automatically superimposes the areas of detected protein spots onto all individual gel images to extract mean intensities of all the corresponding protein spots. The output Excel file is saved in the same folder with all the aligned and cropped gel images. The user can access all output files (mean gel image, segmented gel images, and the Excel data file) in a single folder.

MatGel uses the watershed segmentation algorithm implemented in the MATLAB Image Processing Toolbox to detect the edges of protein spots [7]. The watershed segmentation method represents a gray-scale image as a topological surface with the value of image intensity or brightness at each pixel as heights. The bright pixels represent peaks and dark pixels valleys on the topological surface. Consequently, the boundary of the valley or water basin is the edge of the object [8].

**Fig. 1:**
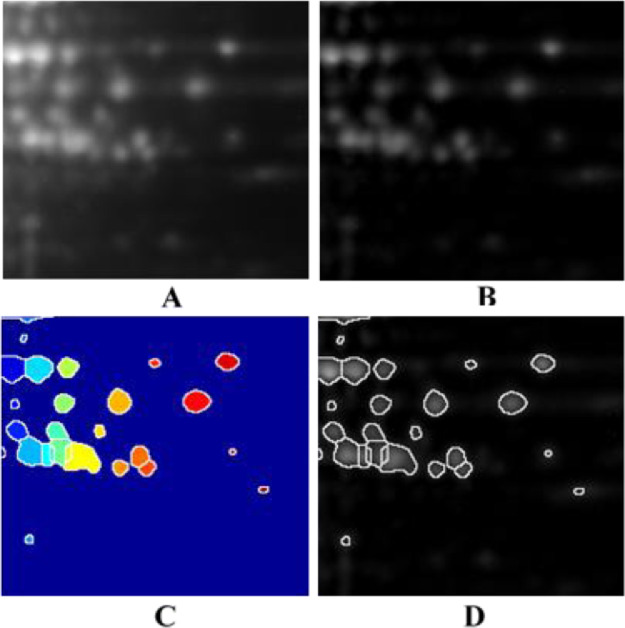
An example of the edge detection and segmentation with MatGel. (A) Original 2D-PAGE gel image (B) TopHat filtered gel image (C) Watershed segmentation (D) Segmented areas overlaid on TopHat filtered gel image.

We have performed the analysis of 2D-PAGE gel images for maize kernel proteins with MatGel. The program provides an easy and highly efficient tool for the detection and quantification of protein spot areas and pixel intensities from an unlimited number of 2D-PAGE gel images. Fig. 1 gives an example showing the edge detection and segmentation of protein spots by using MatGel. The details of the analysis are provided in the Supplementary Material.

MatGel also allows advanced users to make modifications in the current script files based on their specific needs. The followings are helpful hints based on our results. For the image registration ‘cp2tform’ function, ‘nonreflective similarity’ should be used. In the ‘Top Hat Filter step’, ‘disk’ size can be adjusted to improve edge detection. Reducing the size of ‘ROI’ region can improve the detection of low intensity protein spots. The watershed segmentation steps can be adjusted to reduce over segmentation. Lastly, personal communications are welcome for the improvement of the current script files.

## Conclusions

The 2D-PAGE gel technique profiles hundreds of proteins by their unique positions, spot areas, and spot pixel intensities that are resolved in 2D-PAGE gel images. The size of protein spot areas and their pixel intensities are directly related to the protein expression levels. Due to the irregularity in the shapes of protein spots commonly present in 2D-PAGE gel images, large scale quantitative analysis of protein expression levels across multiple 2D-PAGE protein gels has been a challenging issue in proteomic studies. Manual analysis of protein spots through visual comparison is a difficult and time-consuming process. In this research, we have developed a new program, MatGel, for 2D-PAGE gel image analysis. It is simple and highly automated. The output data can be readily used in statistical analysis programs.

## CRediT authorship contribution statement

**Alka Tiwari:** Software, Writing – original draft. **W. Paul Williams:** Conceptualization, Writing – review & editing. **Xueyan Shan:** Conceptualization, Methodology, Software, Writing – original draft.

## Declaration of Competing Interest

The authors declare that they have no known competing financial interests or personal relationships that could have appeared to influence the work reported in this paper.

## Data Availability

Data will be made available on request.

## Appendix Supplementary materials

Image, application 1
