# Supplementary material for: MatGel: A MATLAB program for quantitative analysis of 2D polyacrylamide electrophoresis (2D-PAGE) protein gel images
Source: MethodsX. 2022 Nov 21;9:101930. doi: 10.1016/j.mex.2022.101930 (PMC9703371; doi:10.1016/j.mex.2022.101930)

***Supplementary Material***

**MatGel: A MATLAB program for quantitative analysis of 2D polyacrylamide electrophoresis (2D-PAGE) protein gel images**

Alka Tiwari^1^, W. Paul Williams^2^, Xueyan Shan^1*^

^1^Department of Biochemistry, Molecular Biology, Entomology and Plant Pathology, Mississippi State University, Mississippi State, MS 39762; ^2^USDA-ARS, Corn Host Plant Resistance Research Unit, Mississippi State, MS 39762, * [xs34@msstate.edu](mailto:xs34@msstate.edu)

**Supplemental Figure 1**

**2DE gel image preparation**

**Image alignment & cropping**

**Segmentation**

**Data Output**

***MatGel major steps***

Image formats:

TIFF, JPEG, BMP, JPG, or PNG etc.

Alignment and cropping of a gel image with the standard gel image

Mean gel image construction, edge detection and watershed segmentation


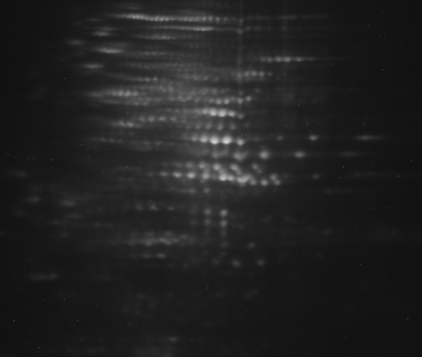

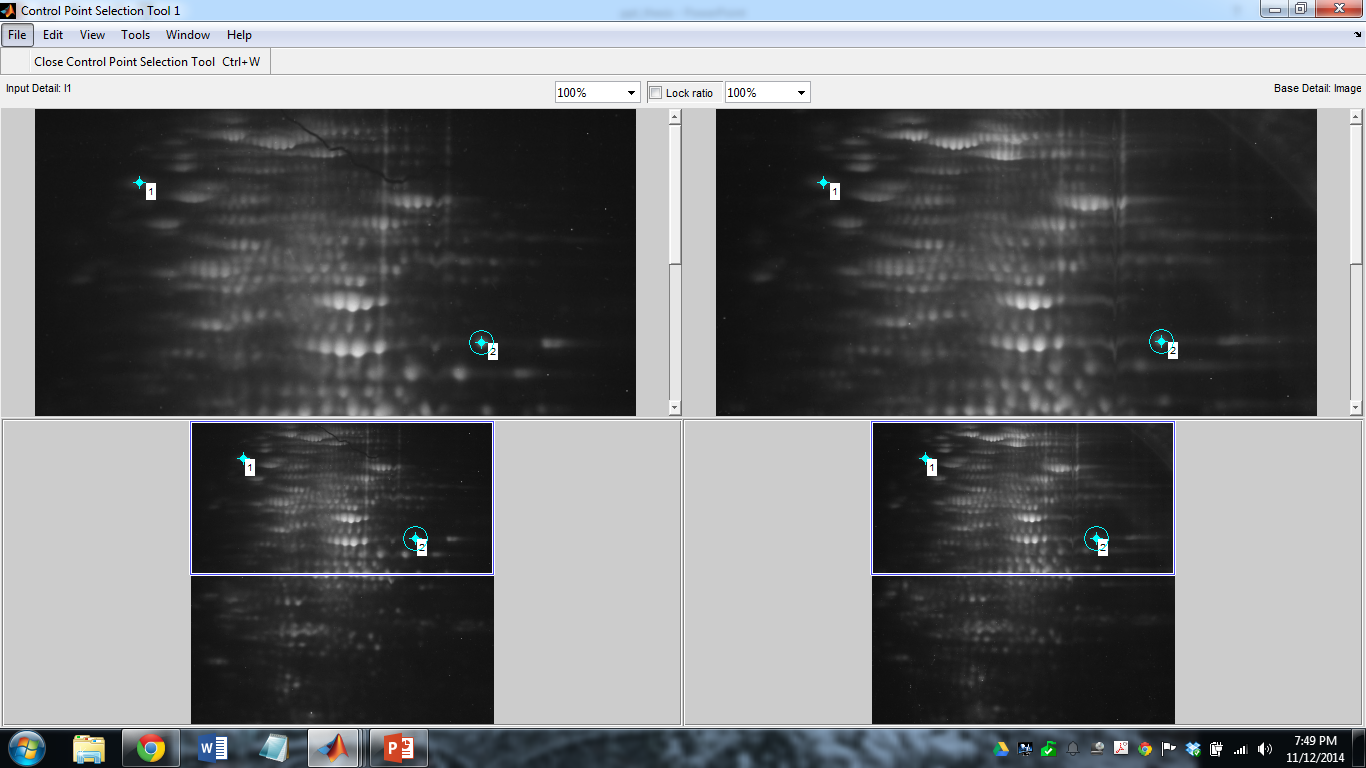

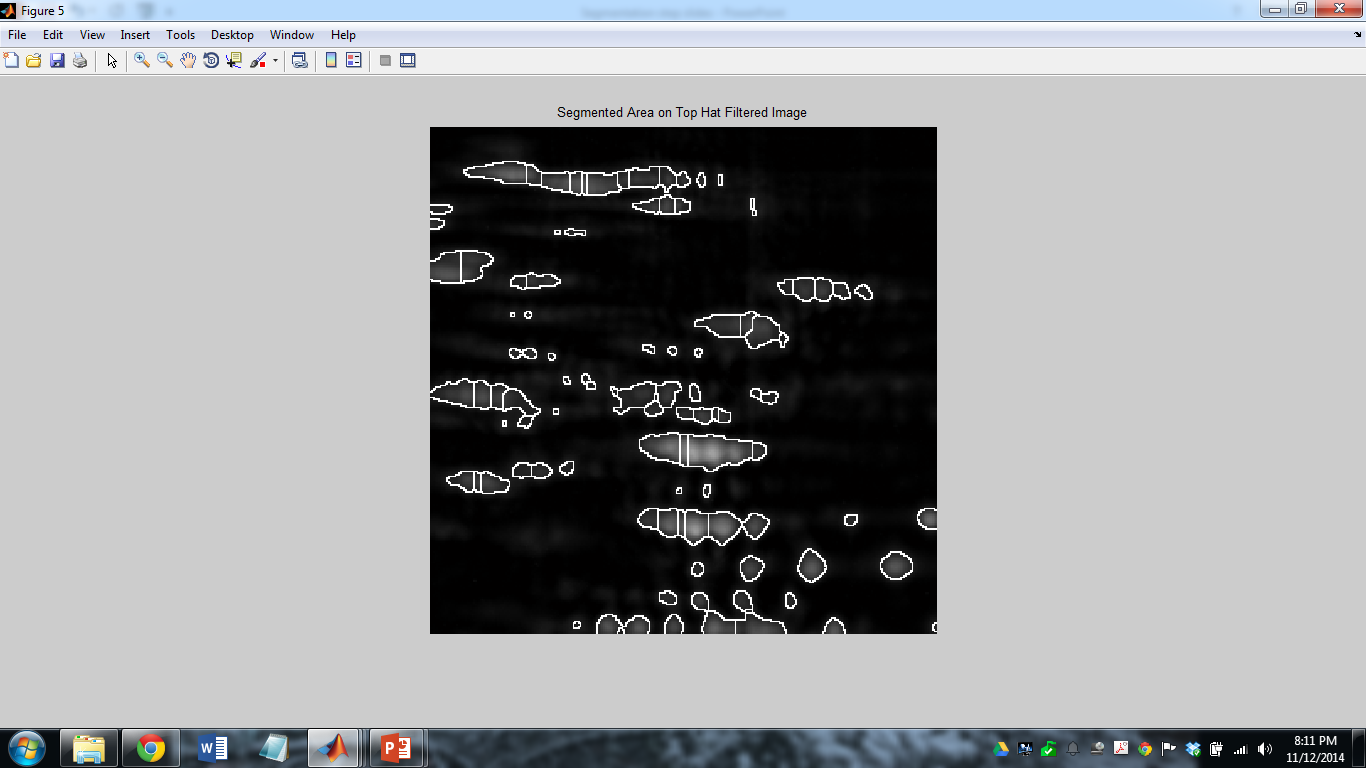


Data are stored in Excel file for further analysis


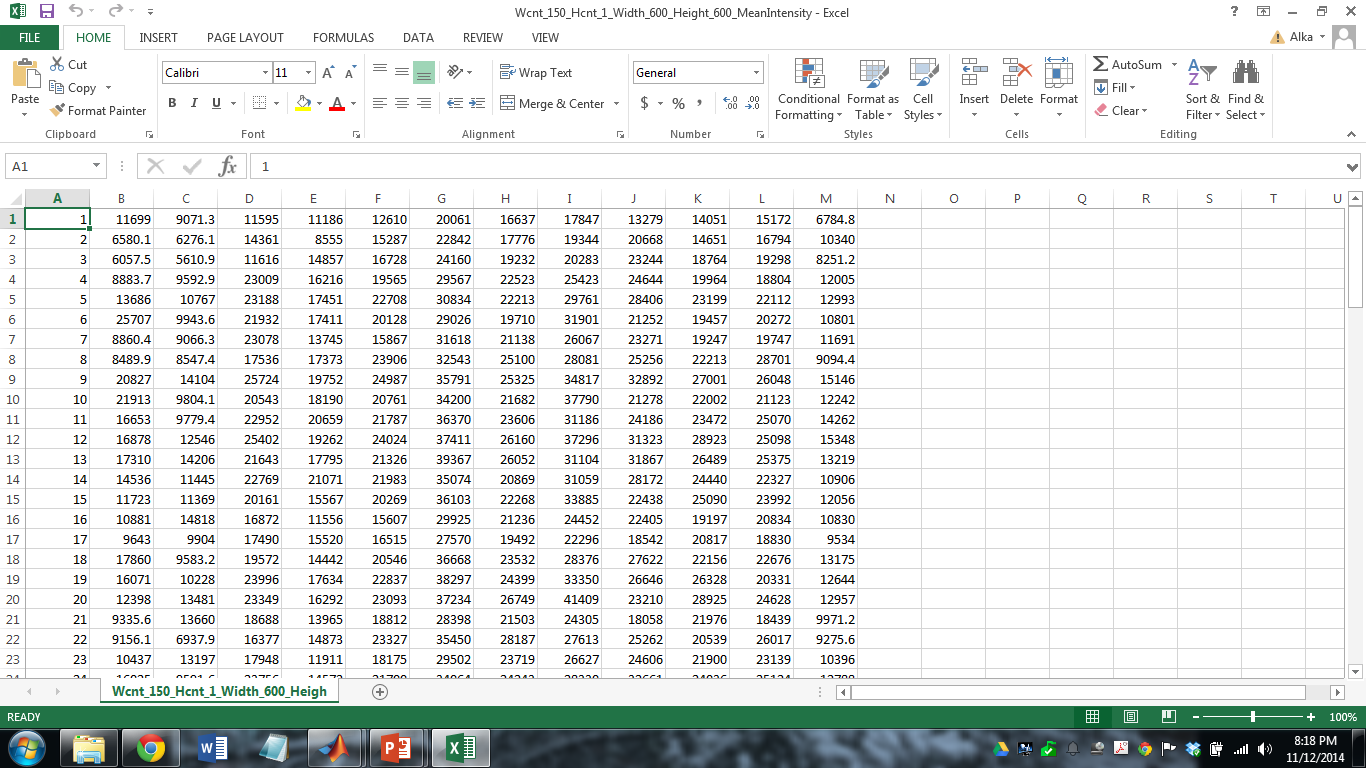


**Supplemental Figure 2**

Load MatGel_align_crop.m file. Click ‘Run’ to execute. Then follow the message box instructions to select a standard gel image and a gel image to be aligned.

**Selection of gel images for the first step of alignment and cropping**


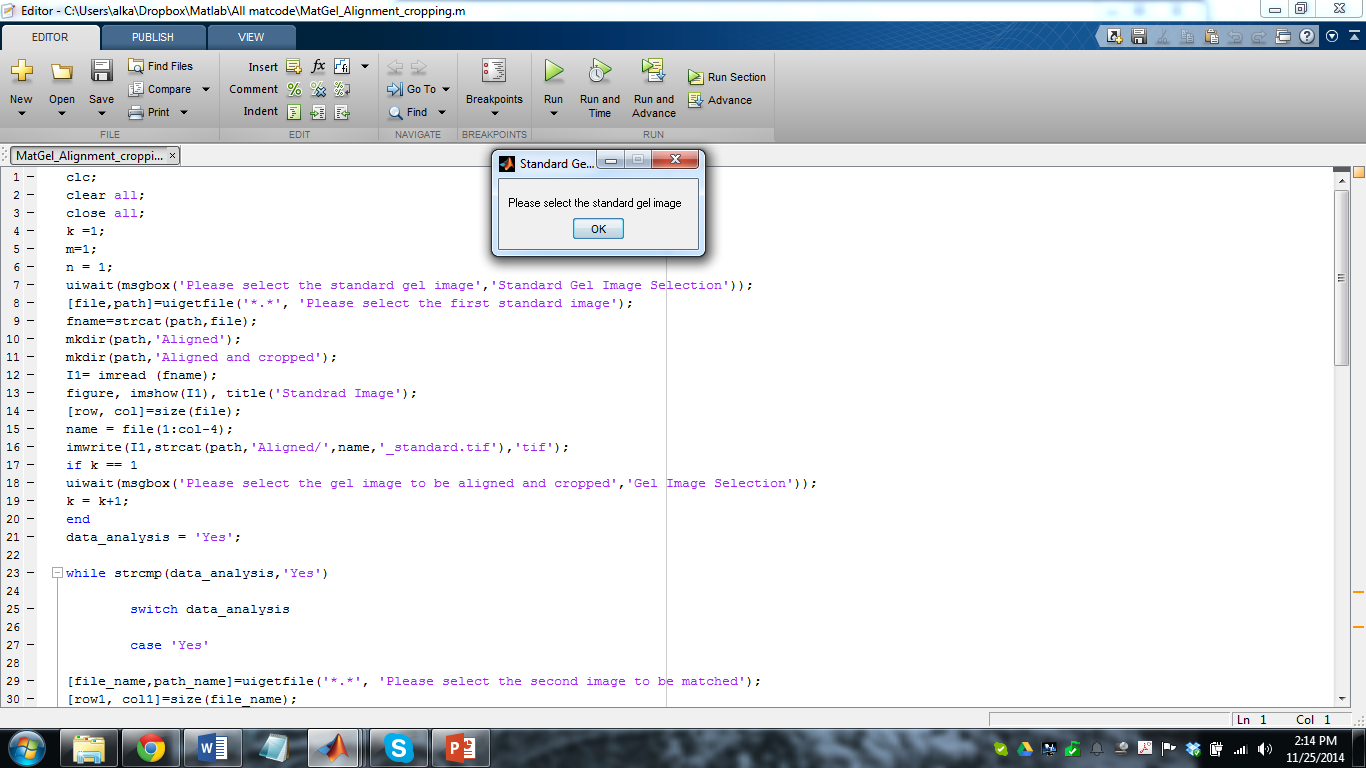


**Supplemental Figure 3**


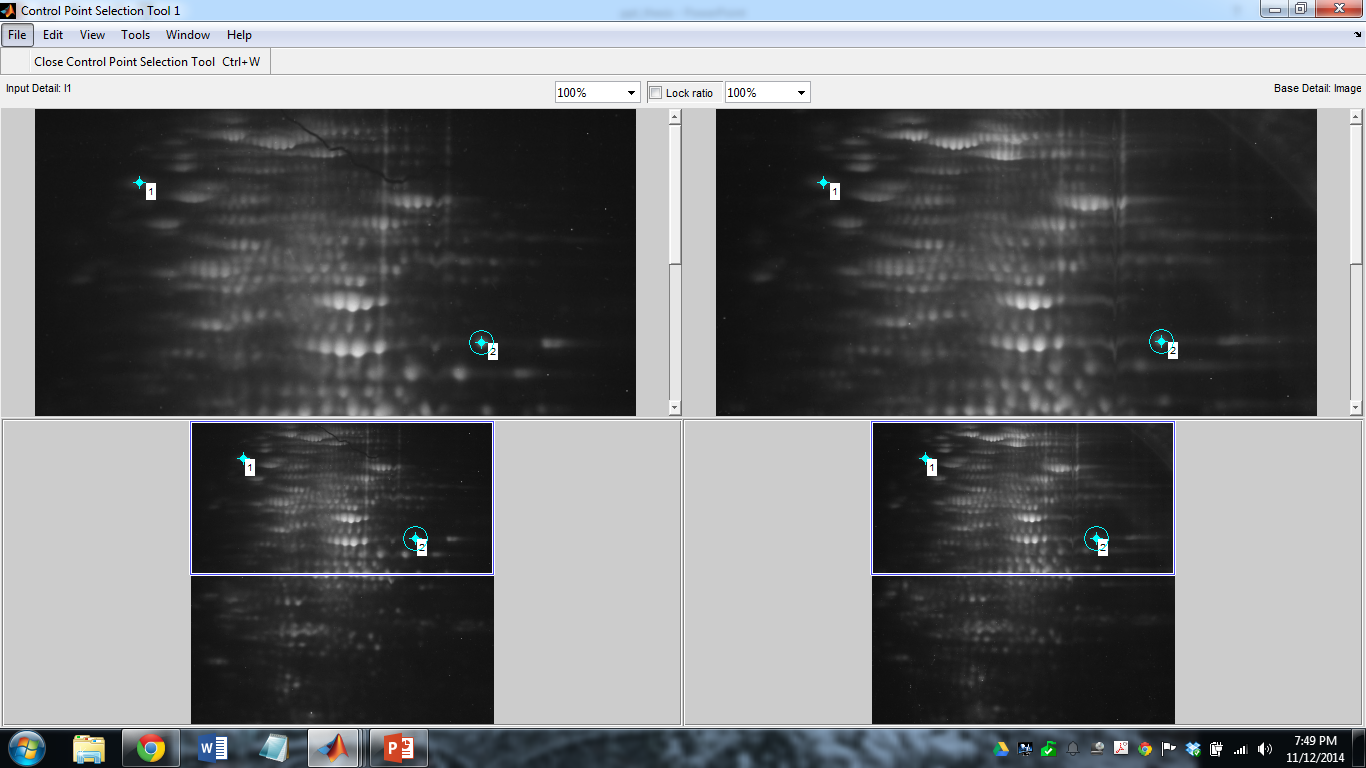


**Control point selection for alignment**

2. Click ‘Close control point selection’ to exit after control points are selected

1. Selected control points

Two control points are selected for each image.

Left image is used as the standard and the right image is to be aligned with the standard.

**Supplemental Figure 4**


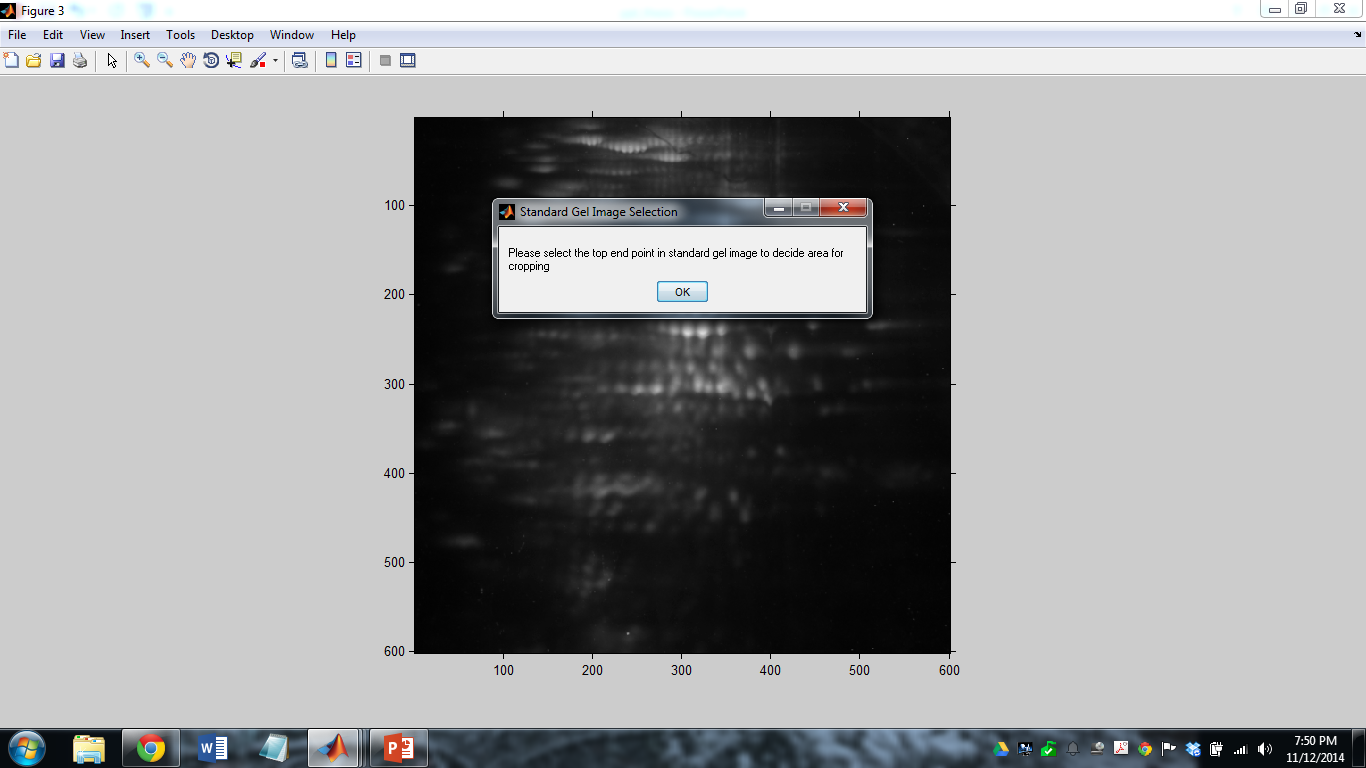


**Image cropping**

This step is requested only one time. The cropping area will be applied to all the gel images.

**Supplemental Figure 5**

A dialog box will appear and ask for the selection of next gel image to be aligned.

**Selection of more gel images for alignment**


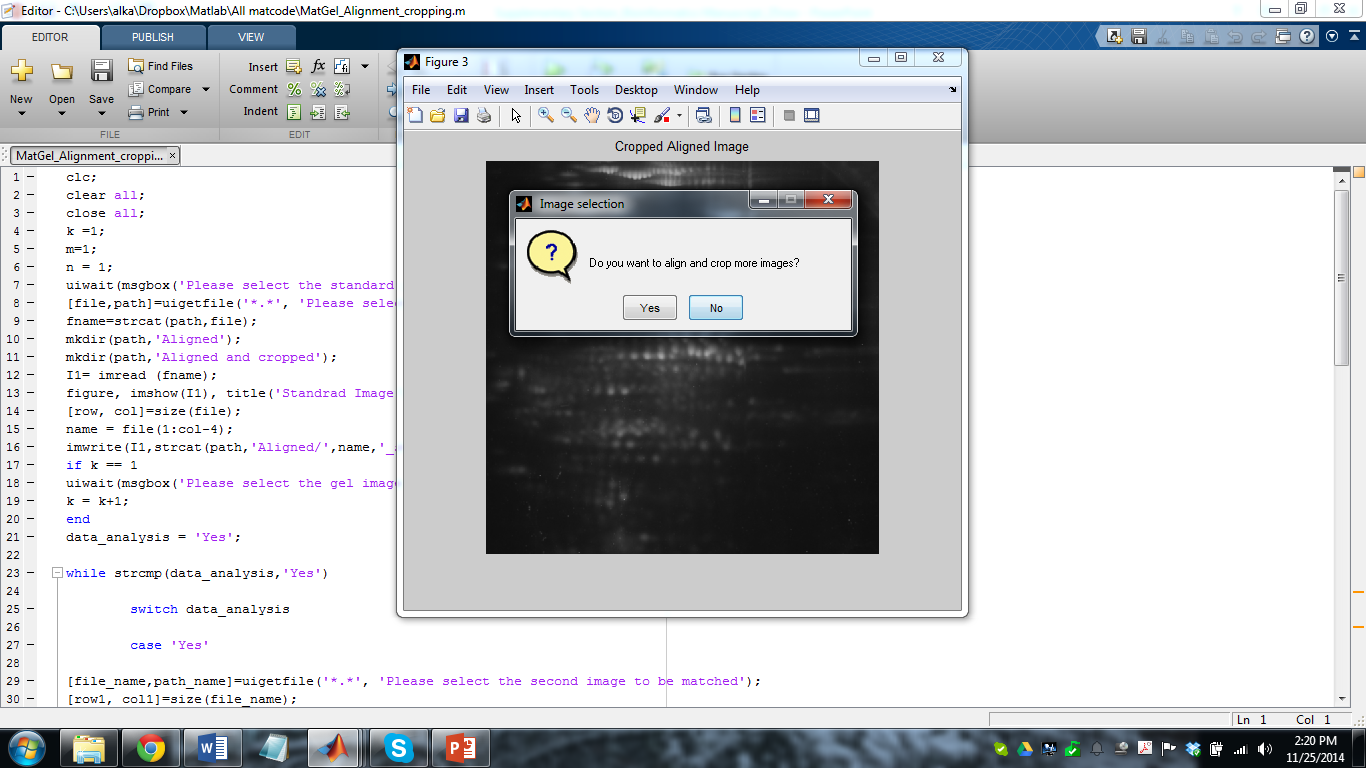


**Supplemental Figure 6**

Launch the ‘MatGel_segmentation.m’ file. Then follow the instruction and select the folder named ‘aligned and cropped’ in the MatGel directory

**Mean gel image construction**


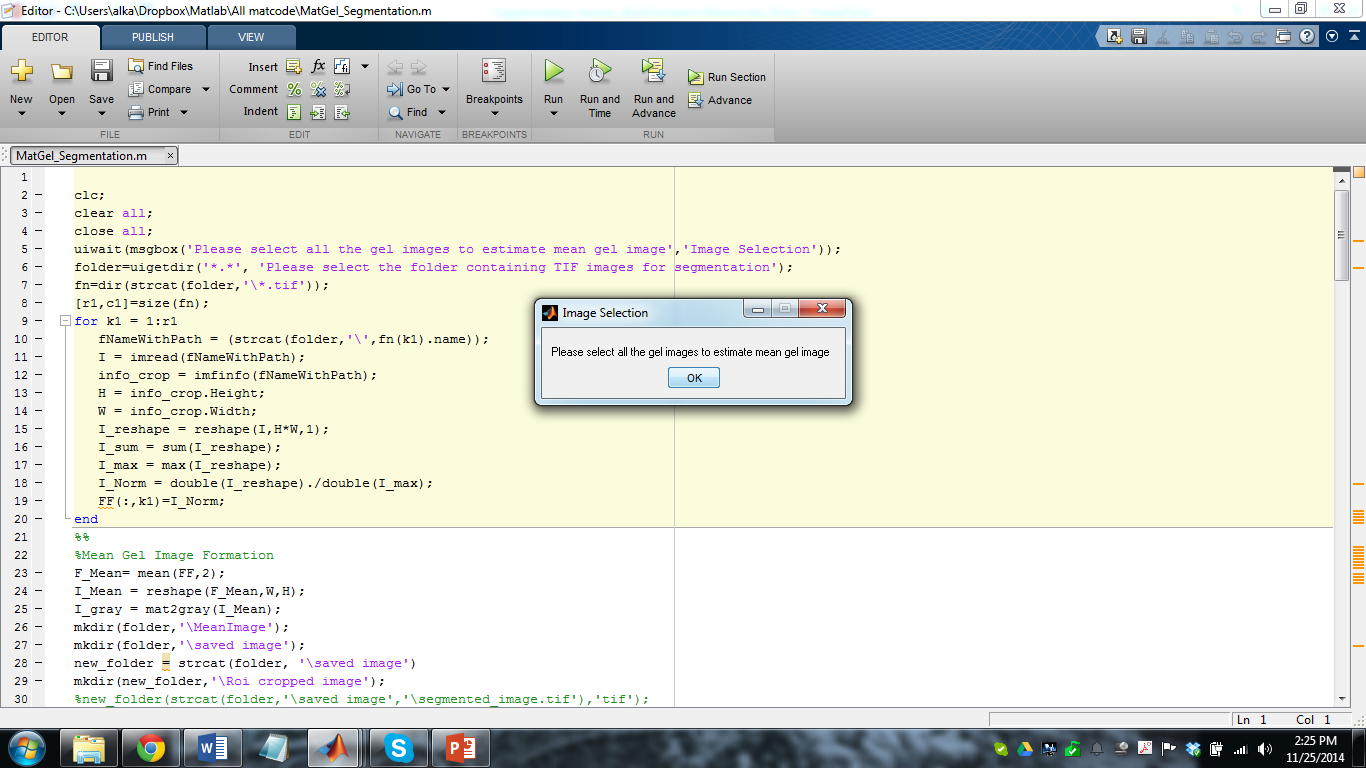


**Supplemental Figure 7**


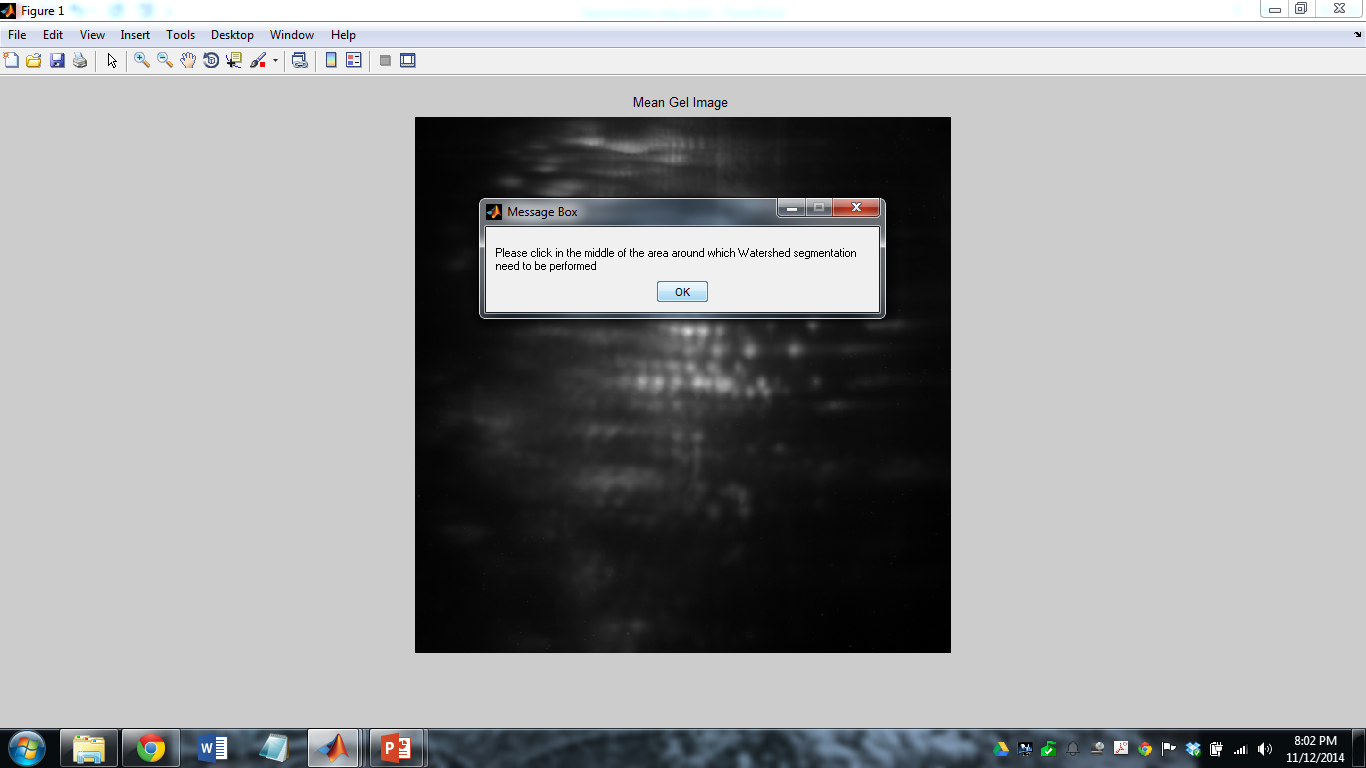


After the mean gel image is constructed, a dialogue box will request the selection of an area (the region of interest) for segmentation.

**The region of interest (ROI) for segmentation**

**Supplemental Figure 8**

**ROI selection continued**

Reducing the size of the ROI area can improve the detection of low intensity protein spots. The example shows a 150 by 150 pixel area of ROI.


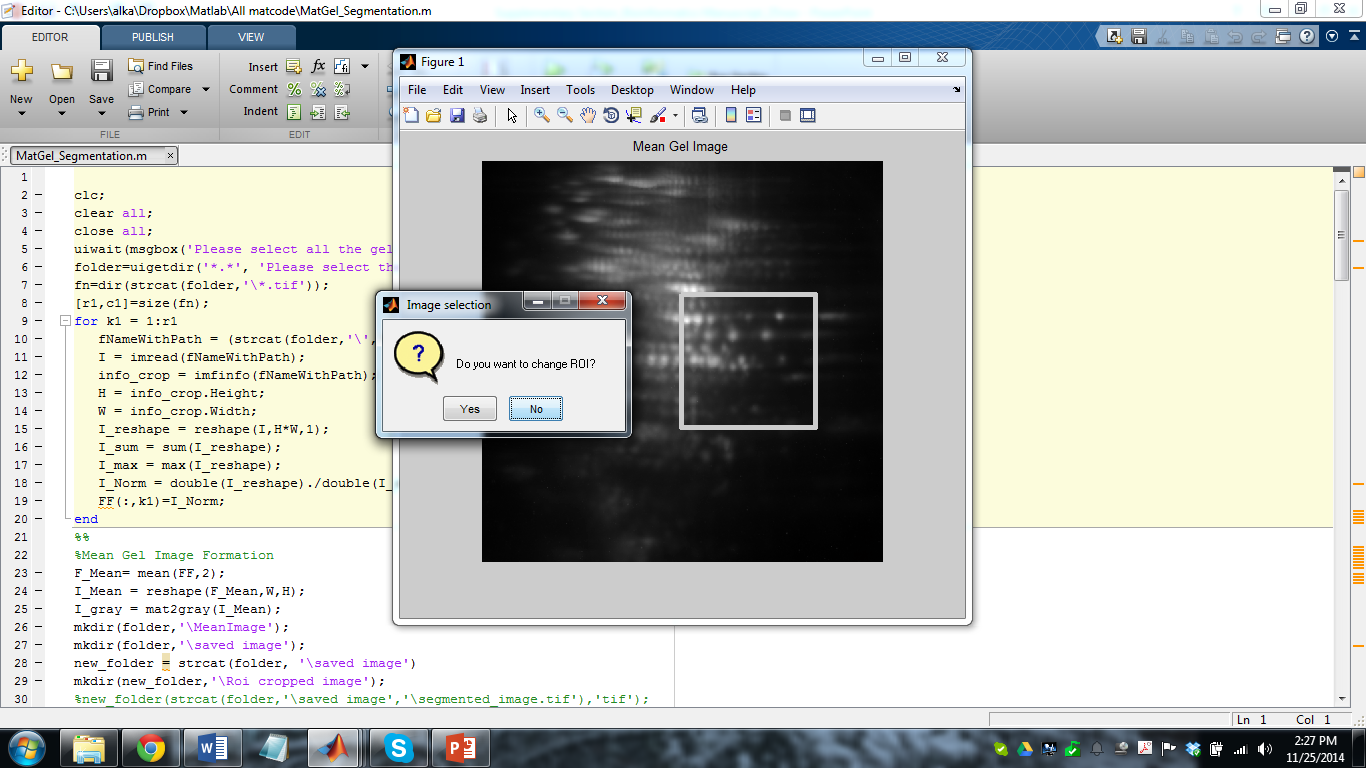


**Supplemental Figure 9**

**Edge detection and segmentation**

Edge detection and segmentation will be performed in the selected ROI area with a size of 150 by 150. This will be followed by the image analysis on all individual images.


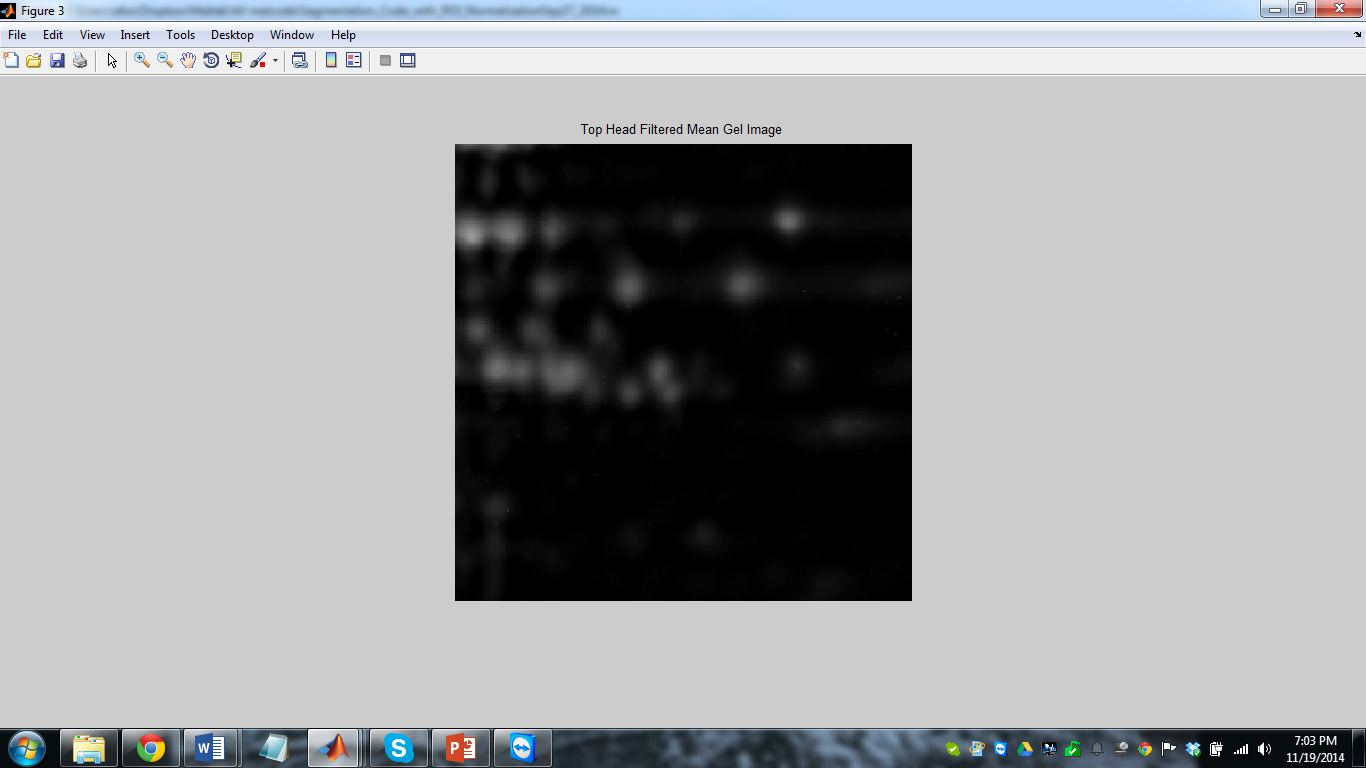


**Supplemental Figure 10**

The image analysis on all individual gel images can be finished within seconds.

**Edge detection and segmentation (continued)**


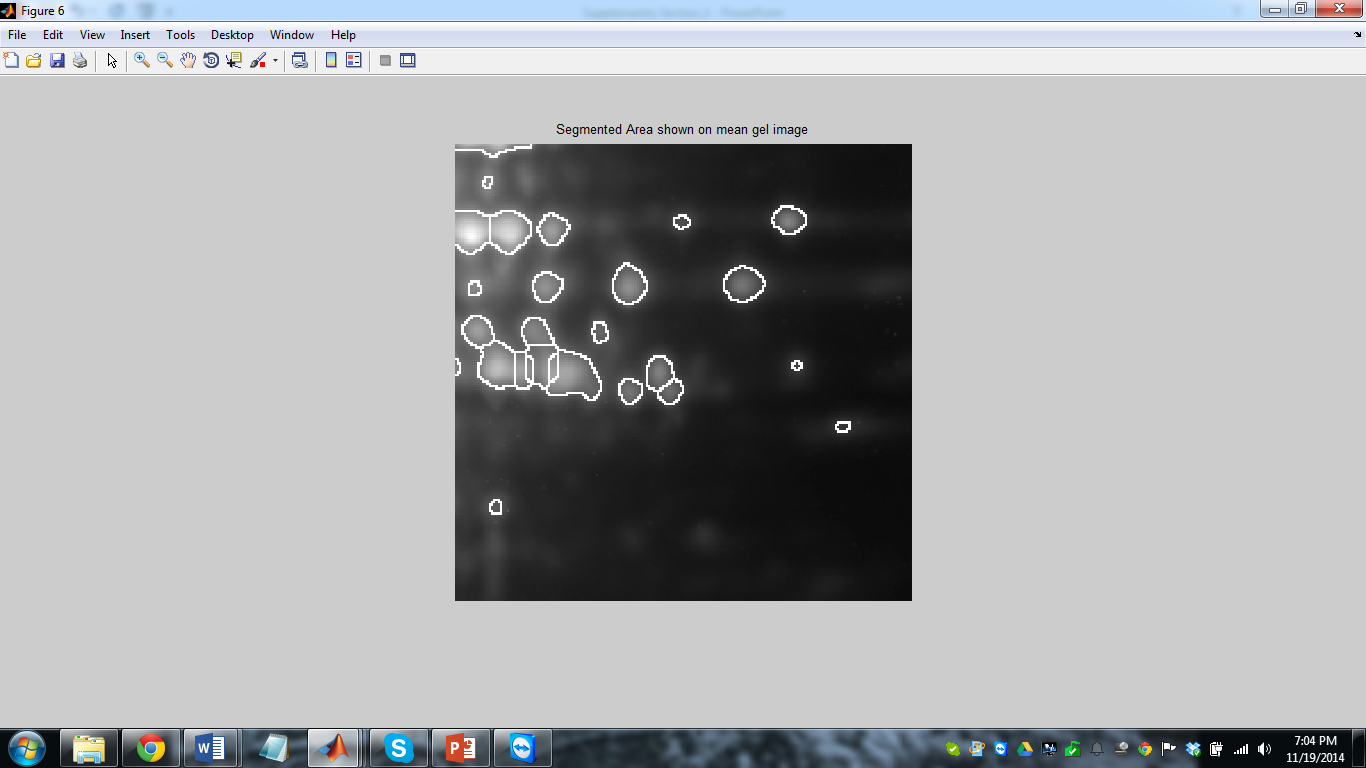

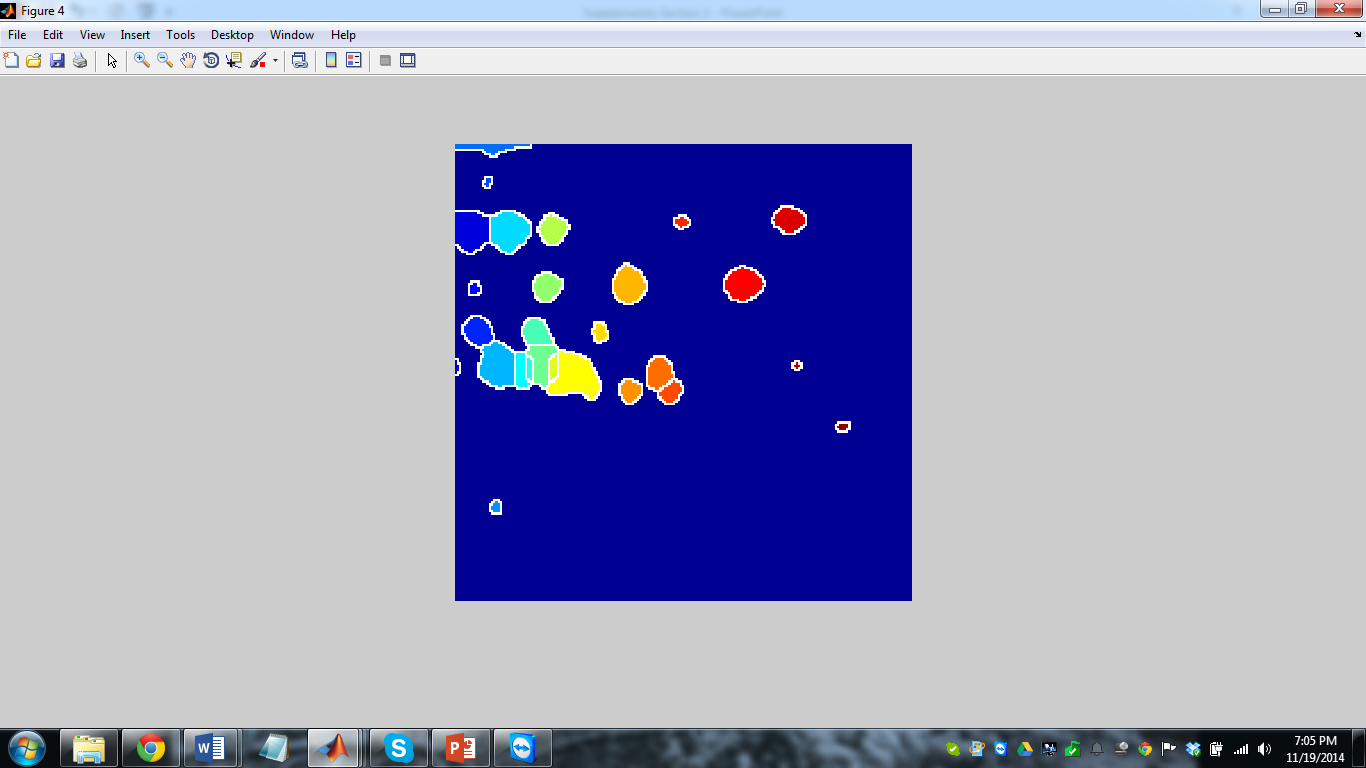

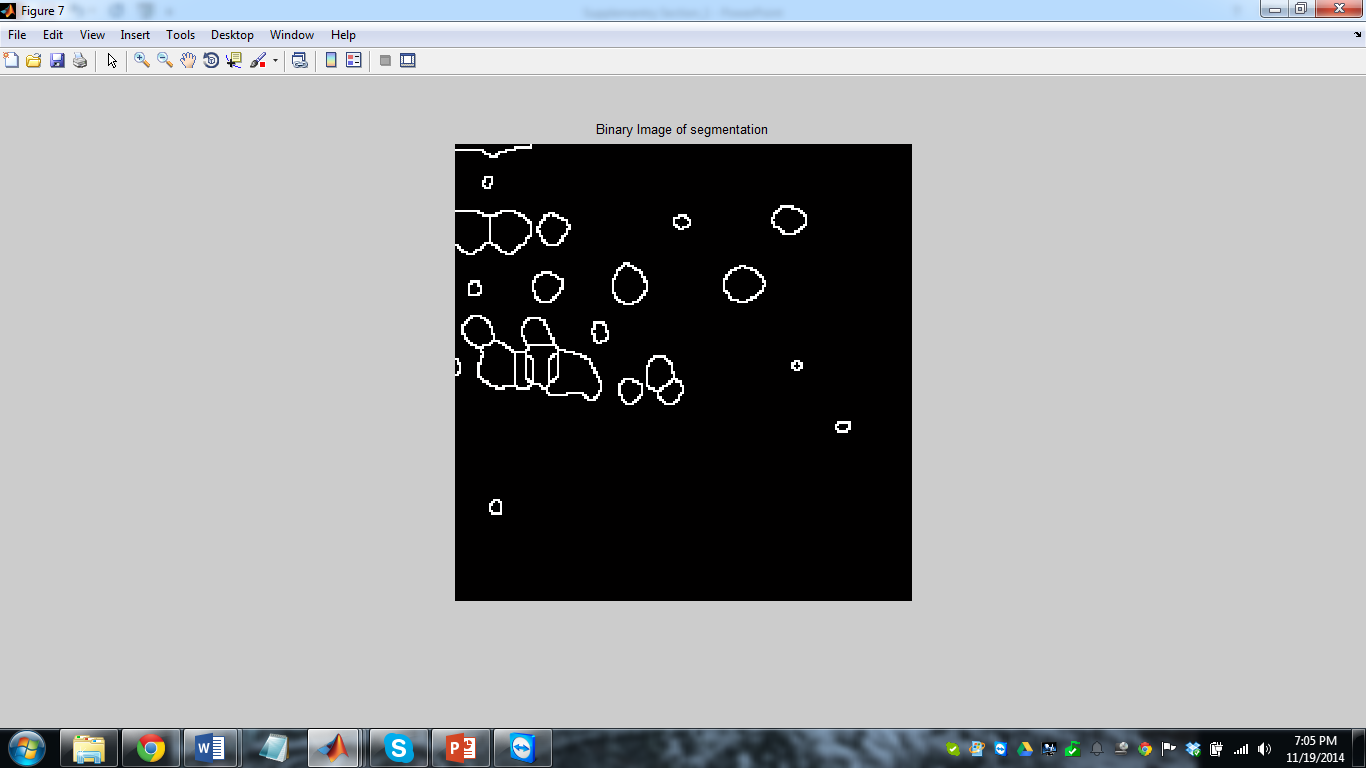

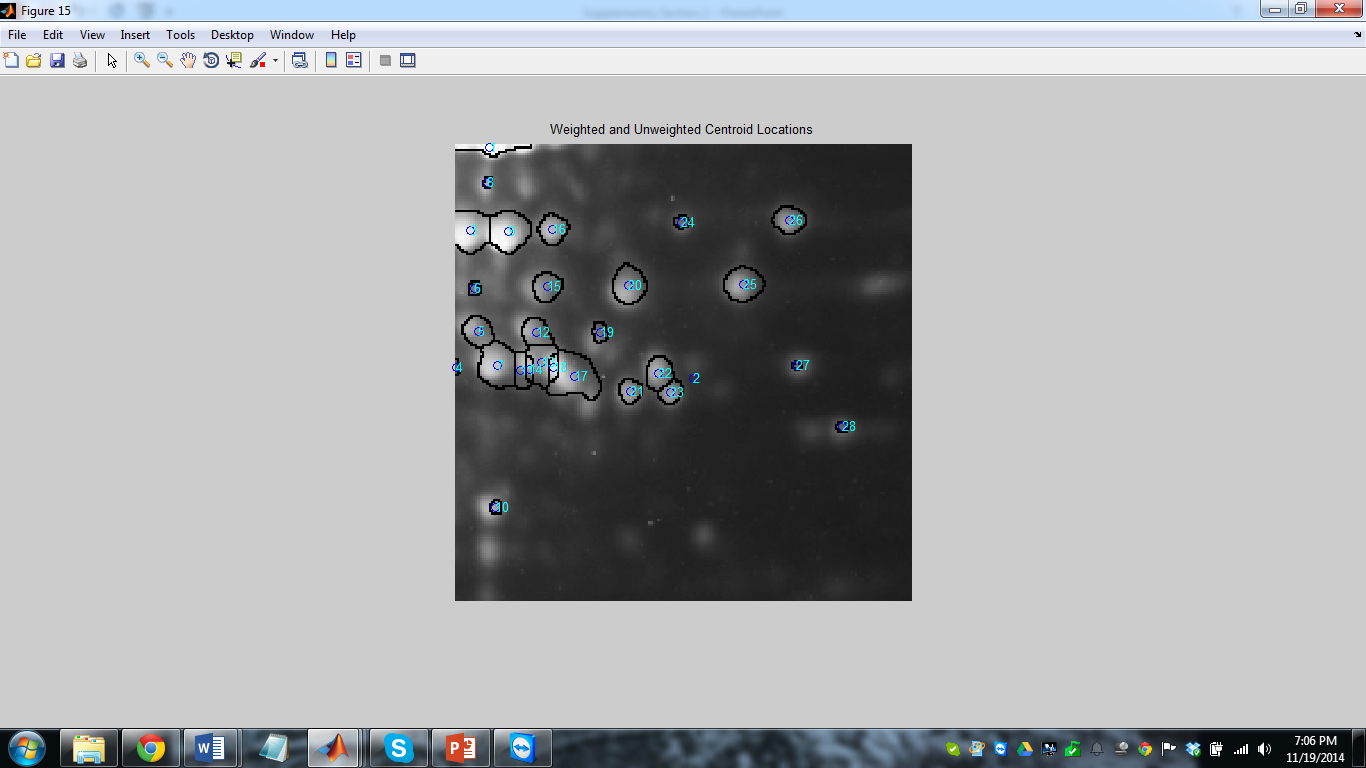


**Supplemental Figure 11**

Once the segmentation is performed, an Excel file is automatically generated containing the area and intensity data of all the detected protein spots and is ready for further statistical analysis.

**Output MatGel data file**


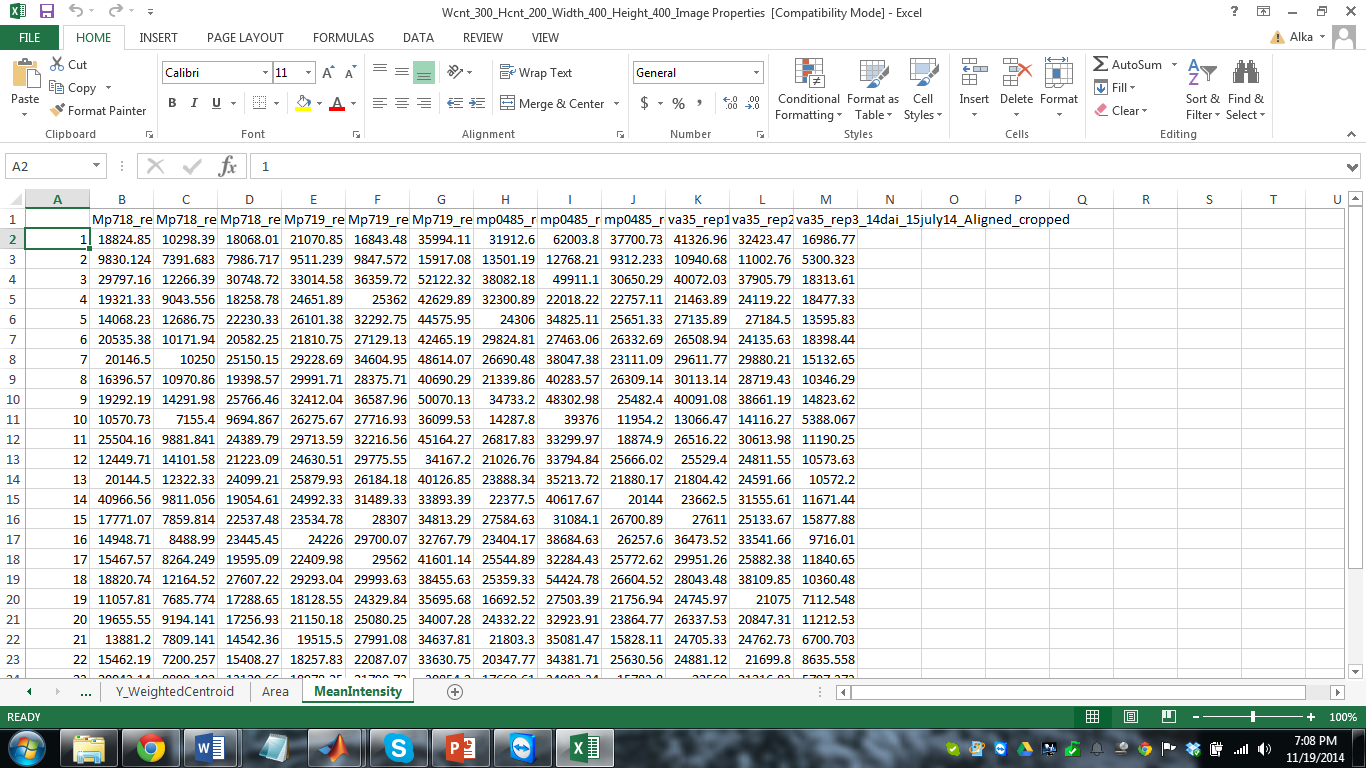

Supplement: Supplementary file 1 [file mmc1.docx]
